# Contrast-Enhanced Ultrasound for Evaluation of High-Intensity Focused Ultrasound Treatment of Benign Uterine Diseases: Retrospective Analysis of Contrast Safety

**DOI:** 10.1097/MD.0000000000000729

**Published:** 2015-04-24

**Authors:** Chong-Qing Cheng, Rui-Tao Zhang, Yu Xiong, Li Chen, Jian Wang, Guo-Hua Huang, Ke-Quan Li, Lian Zhang, Jin Bai

**Affiliations:** From State Key Laboratory of Ultrasound Engineering in Medicine Co-founded by Chongqing and the Ministry of Science and Technology (CQC, RTZ, JW, GHH, KQL, LZ, JB), Chongqing Key Laboratory of Ultrasound in Medicine and Engineering, College of Biomedical Engineering, Chongqing Medical University; Department of Obstetrics and Gynecology (RTZ), Fuling Central Hospital; Department of Obstetrics and Gynecology (YX, LC, KQL), Chongqing Haifu Hospital; Department of Obstetrics and Gynecology (JW), Three Gorges Central Hospital, Chongqing; and Department of Obstetrics and Gynecology (GHH), Suining Central Hospital, Sichuan, China.

## Abstract

As a noninvasive treatment technique, ultrasound-guided high-intensity focused ultrasound (HIFU) has been considered as a routine treatment for uterine fibroids and adenomyosis in China. Contrast-enhanced ultrasound (CEUS) has been proposed as another option to assess the treatment efficacy during HIFU treatment. The aim of this investigation is to evaluate the adverse effects of HIFU ablation for benign uterine diseases in a group of patients studied with ultrasound contrast agent (UCA), in comparison with a group of patients not exposed to UCA. From November 2010 to December 2013, 2604 patients with benign uterine diseases were treated with HIFU. Among them, 1300 patients were exposed to an UCA, whereas 1304 patients were not.

During HIFU procedure, the incidences of leg pain, sacral/buttock pain, groin pain, treatment area pain, and the discomfort “hot” sensation on skin were higher in the patients who were exposed to SonoVue (Bracco, Milan, Italy) than those who were not (20.5% vs 11.7%, 52.5% vs 42.3%, 6.5% vs 4.5%, 68.9% vs 55.4%, and 48.1% vs 42.9%, respectively). Among the postoperative adverse effects, the incidence of lower abdominal pain was significantly higher in patients who were exposed to an UCA than those who were not (51.2% vs 39.9%, *P* < 0.05). Two patients who were exposed to an UCA had acute renal function failure.

In conclusion, UCA may increase the incidences of some common HIFU-related adverse effects during HIFU treatment for benign uterine diseases, but most of which were acceptable and self-limited. After HIFU treatment, renal function should be monitored in patients with a history of hypertension or taking nonsteroidal anti-inflammatory drugs.

## INTRODUCTION

As a noninvasive technique, ultrasound-guided high-intensity focused ultrasound (USgHIFU) has been widely used in treating different types of solid tumors.^1–4^ In comparison with magnetic resonance imaging-guided high-intensity focused ultrasound (MRgHIFU), USgHIFU has wider indications, a lower cost, and a shorter treatment time than that of MRgHIFU.^5,6^ Many studies have confirmed that the long-term symptom relief of patients with uterine fibroids or adenomyosis is related to the nonperfused volume (NPV) ratio.^7,8^ Thus, if the NPV ratio is low, the future retreatment rate may be higher, which may cause a greater failure rate and an increased probability of complications. Although it has been confirmed that the ultrasound massive gray-scale changes are reliable in monitoring the response to HIFU, the massive gray-scale changes were not present in around 20% of tumors and may have been transient.^9^ Therefore, intraprocedural contrast-enhanced ultrasound (CEUS) has been proposed as another option to assess the treatment results.^10,11^

Over the last 10 years, CEUS has been widely used in tumor diagnosis or follow-up after local treatment.^12,13^ Recently, intraprocedural CEUS was used to evaluate the therapeutic results of radiofrequency ablation and HIFU treatment.^14,15^ Many studies have demonstrated that the ultrasound contrast agent (UCA)-related adverse effect rates were at a very low level^16–18^; however, a “black-box warning” was required by the US Food and Drug Administration after 4 deaths occurred through the usage of an UCA of Definity (Lantheus Medical Imaging, North Billerica, MA).^19^ In addition, it is different from diagnosis or other kinds of treatment; all patients with uterine fibroids or adenomyosis who take HIFU treatment are requested to have specific bowel preparation before HIFU treatment.^7^ As all patients were advised to ingest liquid food for 2 days, drink bowel preparation solution (1 L of polyethylene glycol electrolyte solution), and have at least a 12-hour fast, some patients may have had dehydration. Although Peng et al^9^ have demonstrated that SonoVue (Bracco, Milan, Italy) can be safely used to assess the therapeutic results, and Jiang et al^20^ showed that SonoVue can be safely used to enhance the ablation effects of HIFU, the sample size in both the studies was relatively small. Therefore, the aim of this retrospective study was to evaluate the safety of intraprocedural CEUS in HIFU treatment for uterine fibroids and adenomyosis.

## METHODS

### Patients

This retrospective study was approved by the ethics committees at our institutions. Every patient signed an informed consent before HIFU treatment.

From November 2010 to June 2013, a total of 2604 patients with uterine fibroids or adenomyosis were treated with HIFU in Chongqing HIFU Hospital, Chongqing Fuling Hospital, Chongqing Three Gorges Central Hospital, Sichuan Suining Central Hospital, and Hunan Xiangya Hospital of Central South University; 1663 patients with symptomatic uterine fibroids and 941 patients with adenomyosis were in this study. During HIFU treatment, 1304 patients were exposed to SonoVue, whereas 1300 patients were not exposed to.

Table 1 shows the baseline characteristics of the patients. There was no significant difference between the 2 groups in age, uterine position, location of lesions, and the size of lesions.

**TABLE 1:**
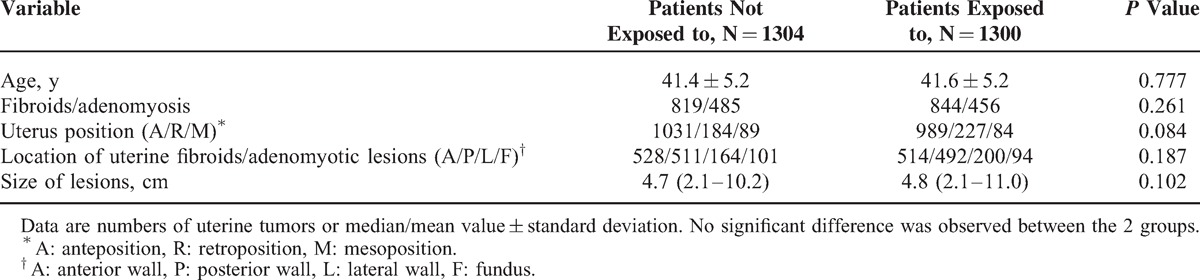
Characteristics of Patients Exposed or Not Exposed to SonoVue

### Pretreatment Preparation

Every patient underwent a pretreatment magnetic resonance imaging (MRI) with a standardized protocol to help confirm the uterus position, size, and location. The pretreatment preparation has been described previously.^1,3^ Briefly, all patients ingested liquid food for 2 days. On the day prior to the treatment, every patient drank a single dosage of bowel preparation solution (1 L of polyethylene glycol electrolyte solution). Following a 12-hour fast, enema was performed in the morning of the treatment day. All patients were requested to shave, degrease, and degas the abdominal skin before HIFU treatment. To optimize the therapeutic acoustic pathway, a urinary catheter was inserted to control the bladder volume with a saline injection. A degassed water balloon was prepared for each patient with the purpose of compressing and pushing bowels away from the acoustic pathway.

### HIFU Treatment

HIFU ablation was performed with the JC HIFU system (Chongqing Haifu Technology, Chongqing, China) equipped with an ultrasound device for real-time guidance of the treatment. The ultrasound beam was produced by a 20 cm diameter transducer with a focal length of 15 cm, operated at a frequency of 0.8 MHz. A 1.0 to 8.0 MHz diagnostic US probe (MyLab 70; Esaote, Genova, Italy) located at the center of the therapeutic transducer was used to monitor the treatment.

Every patient was carefully positioned prone on the HIFU table. The anterior abdominal wall was in contact with degassed water. HIFU treatment was performed under intravenous conscious sedation. The patients received fentanyl 50 to 400 μg and midazolam hydrochloride 1 to 4 mg to reduce discomfort and prevent movement. They were asked to report any discomfort to an assisting nurse during the procedure; 350 to 400 watts of sonication power was delivered to the target, with the sonication terminating when the gray-scale change at the target region was observed. Patients were discharged from the HIFU unit 30 minutes after HIFU treatment.

### CEUS Examination

CEUS was performed using SonoVue (Bracco). This microbubble agent was first reconstituted in a 25 mg vial with the addition of 5 mL of normal saline. All the patients in the SonoVue group received a bolus containing 2.0 mL of SonoVue solution by injection, followed by a 5 mL saline injection through a hand vein before HIFU ablation. Immediately following HIFU ablation, the patients each had a 1.5 mL of SonoVue solution injected to assess the ablation results. After HIFU treatment, if any unexpected residual lesion was spotted in the treated lesion, an additional HIFU ablation in the same session of HIFU treatment can be performed.

### Follow-Up

To evaluate the effectiveness of the ablation results, a post-HIFU MRI was performed 1 day after the HIFU procedure. The lesion volume and the NPV were measured. The fractional ablation (defined as NPV divided by the lesion volume) was also calculated. The adverse effects and complications during and after HIFU treatment were recorded.

### Statistical Analysis

Data was reported as a mean ± standard deviation or median value, and analysis was performed using SPSS software (SPSS19, IBM SPSS, Chicago, IL). A nonparametric Mann–Whitney test was used for statistical comparisons of age, the size of tumors, the uterine fibroids/adenomyosis volume, NPV, fractional ablation, total sonication time, sonication time for 1 cm^3^, and average total energy and energy efficiency factor (EEF) (with vs without SonoVue). The χ^2^ test helped compare rates of lesion sites, lesion types, the rates of massive gray-scale changes, and incidence rates of adverse effects or complications (with vs without SonoVue). A *P* value <0.05 was an indication of significant difference.

## RESULTS

### Demographic Characteristics of the Patients

Table 1 shows the characteristics of the patients. Among the 1304 patients who were not exposed to SonoVue, 819 patients were diagnosed as having uterine fibroids and 485 patients with adenomyosis; anteposition of the uterus was found in 1031 patients, retroposition of the uterus was seen in 184 patients, and mesoposition of the uterus was found in 89 patients. Of them, 528 had the lesions located at the anterior wall of the uterus, 511 patients had the lesions at the posterior wall of the uterus, 164 of them had lesions at the lateral wall of the uterus, and 101 patients had the lesions at the fundus. The median size of the fibroids/adenomyotic lesions was 4.7 (range, 2.1–10.2) cm in diameter.

Out of the 1300 patients who were exposed to SonoVue, 844 patients were diagnosed as having uterine fibroids, whereas 456 were having adenomyosis; anteposition of the uterus was observed in 989 patients, retroposition of the uterus was seen in 227 patients, and mesoposition of the uterus was observed in 84 patients. Of them, 514 patients had the lesions at the anterior wall of the uterus, 492 patients had the lesions at the posterior wall of the uterus, 200 patients had the lesions at the lateral wall of the uterus, and 94 of them had the lesions at the fundus of the uterus. The median size of the uterine fibroids/adenomyotic lesions was 4.8 (range, 2.1–11.0) cm. No significant difference was observed in baseline characteristics between the 2 groups.

### Postprocedure Evaluation

All patients completed HIFU treatment in this study. In patients with uterine fibroids, the median fibroid volume and the median NPV in patients who were not exposed to SonVue were 57.4 and 40.9 cm^3^, respectively; the rate of massive gray-scale changes for fibroids in patients who were not exposed to SonoVue was 60.8%(498/819); the median fractional ablation for fibroids in patients who were not exposed to SonoVue was 79.0%; the median sonication time for ablation of 1 cm^3^ of fibroid volume was 19.3 s/cm^3^; the median total energy used to ablate uterine fibroids was 338275.0 J; and the median EEF was 8.0 J/mm^3^. Although the median fibroid volume and the median NPV in patients exposed to SonVue were 57.2 and 42.7 cm^3^, respectively, the rate of massive gray-scale changes for fibroids in patients who were exposed to SonoVue was 61.1% (516/844), the median fractional ablation for fibroids in patients exposed to SonoVue was 83.1%, the median sonication time for ablation of 1 cm^3^ of fibroid volume was 21.3 s/cm^3^, the median total energy used to ablate uterine fibroids was 334000.0 J, and the median EEF was 8.3 J/mm^3^. No significant difference was observed except the fractional ablation between the 2 groups (Table 2).

**TABLE 2:**
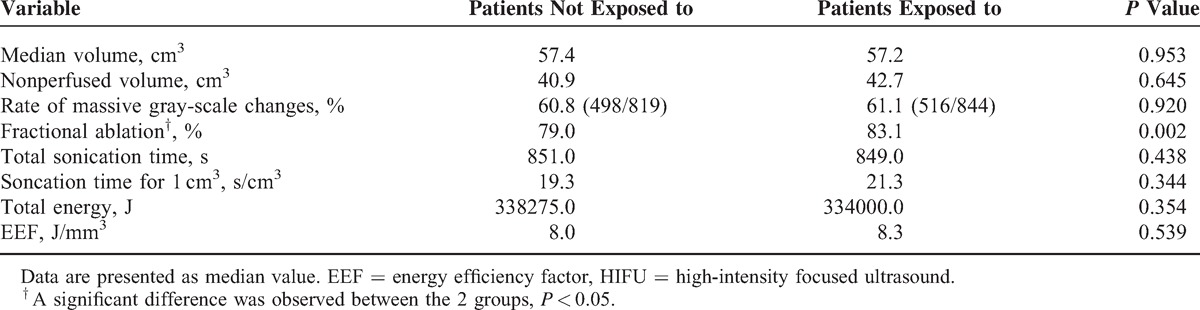
Comparison of HIFU Treatment Results of Patients With Uterine Fibroids Who Were or Were Not Exposed to SonoVue

Table 3 shows that the median volume of adenomyotic lesions and the average NPV in patients who were not exposed to SonoVue were 51.2 and 32.9 cm^3^, respectively; the rate of massive gray-scale changes for adenomyotic lesions in patients who were not exposed to SonoVue was 66.4% (322/485); the median fractional ablation for adenomyotic lesions in patients who were not exposed to SonoVue was 72.6%; the median sonication time for ablation of 1 cm^3^ of adenomyotic lesion volume was 24.2 s/cm^3^; the median total energy used to ablate adenomyotic lesions was 280470.0 J; and the median EEF was 9.1 J/mm^3^. Although the median adenomyotic lesion volume and the median NPV in patients exposed to SonoVue were 64.7 and 39.4 cm^3^, respectively, the rate of massive gray-scale changes for adenomyotic lesions in patients who were exposed to SonoVue was 72.6% (331/456), the median fractional ablation for adenomyotic lesions in patients were exposed to SonoVue was 70.4%, the median sonication time for ablation of 1 cm^3^ of adenomyotic lesion volume was 23.2 s/cm^3^, the median total energy used to ablate adenomyotic lesion was 390550.0 J, and the median EEF was 8.6 J/mm^3^. Significant difference was observed between the 2 groups in median volume of the adenomyotic lesion, NPV, total sonication time, and total energy used.

**TABLE 3:**
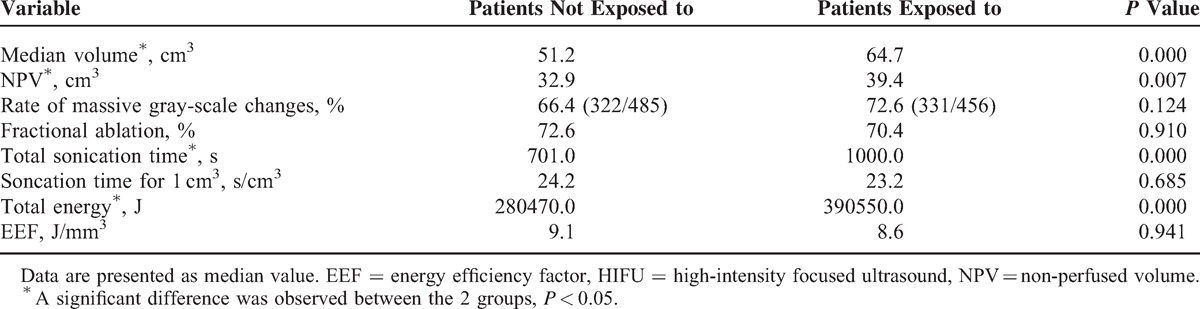
Comparison of HIFU Treatment Results of Patients With Adenomyosis Who Were or Were Not Exposed to SonoVue

### Intraoperative Adverse Effects

Table 4 shows that 11.7% (153/1304) of the patients who were not exposed to SonoVue complained of transient leg pain during HIFU sonication, whereas 20.5% (266/1300) of patients who were exposed to SonoVue reported this pain. Sacrum pain was presented in 42.3% (551/1304) of the patients who were not exposed to SonoVue and 52.5% (682/1300) of the patients who used SonoVue for evaluation during HIFU. Groin pain was reported in 4.5% (59/1304) of patients who did not use SonoVue and was seen in 6.5% (85/1300) of patients who used SonoVue. Pain in the treated region was reported in 55.4% (723/1304) of patients who were not exposed to SonoVue and in 68.9% (896/1300) of patients who were exposed to SonoVue. The discomfort “hot” skin sensation was reported in 42.9% (559/1304) of patients who were not exposed to SonoVue and 48.1% (625/1300) in patients who used SonoVue.

**TABLE 4:**
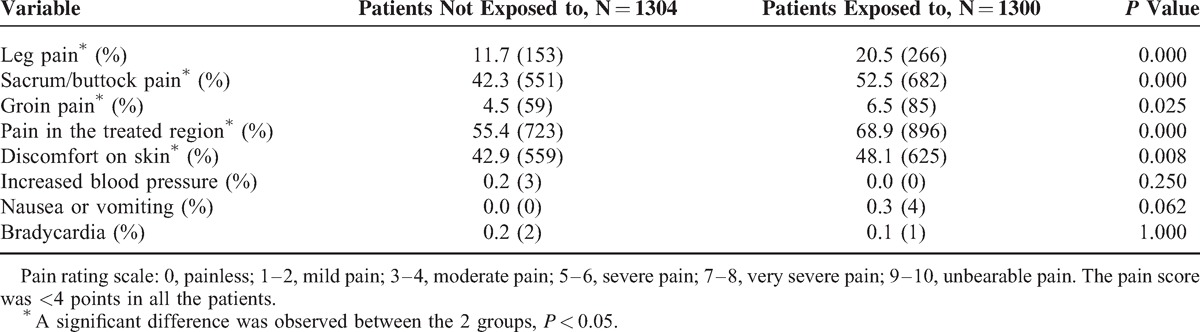
Comparison of Intraprocedure Incidence Rates of Adverse Effects in Patients Who Used With Patients Who Did Not Use SonoVue

### Postoperative Adverse Effects and Complications

Table 5 shows that the incidence rate of lower abdominal pain after HIFU treatment was significantly higher in patients who were exposed to SonoVue than those who were not (*P* < 0.05). But no significant difference in the incidence rates of sacrum pain, leg pain, groin pain, nausea or vomiting, fever, skin blisters, facial edema, and rash and skin itch between the 2 groups was recorded. In this study, we found that 2 patients in those who were exposed to SonoVue had acute renal function failure, but no such case was found in the patients who were not exposed to SonoVue. Most of the adverse effects subsided 1 week after HIFU treatment. With regard to the 2 cases involving acute renal function failure, the renal function fully recovered after renal dialysis.

**TABLE 5:**
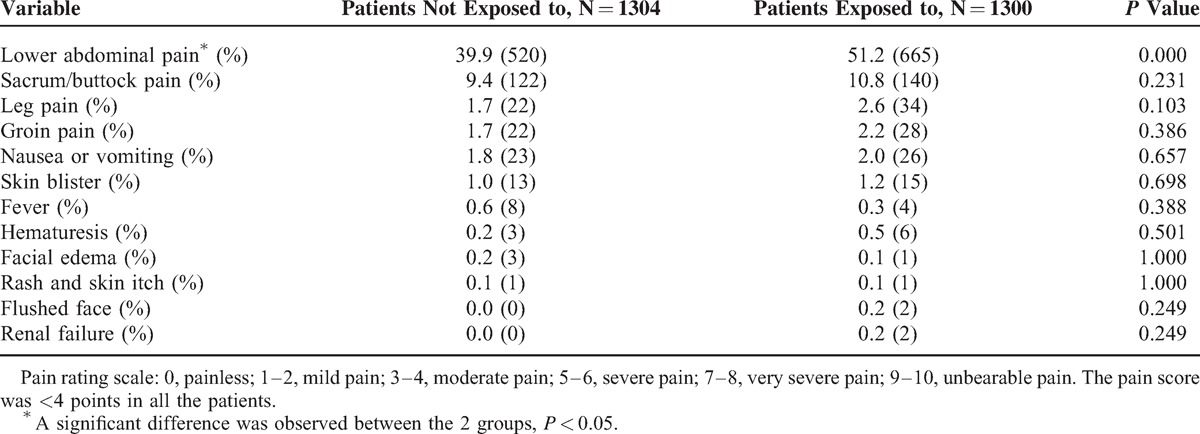
Comparison of Incidence Rates of Postprocedure Adverse Effects and Complications in Patients Who Were With Patients Who Were Not Exposed to SonoVue

## DISCUSSION

Over the last 10 years, HIFU has been widely used in the treatment of uterine fibroids and adenomyosis.^21,22^ Many studies have demonstrated that HIFU treatment for uterine fibroids and adenomyosis is safe and effective.^23–25^ Based on the classification of the Society of Interventional Radiology, the HIFU-related adverse effects or complications that occurred during and/or after HIFU treatment are mild. In fact, the complications disappeared in a short time without any specific treatment. In this present study, our results also showed that HIFU treatment for uterine fibroids or adenomyosis is safe.

SonoVue (Bracco) is the only approved and available ultrasound-enhanced contrast agent in China. In general, SonoVue is a safe UCA with a low incidence of complications. Piscaglia and Blondi^26^ presented their results of abdominal ultrasound examinations from 23,188 patients who used SonoVue, and no fatal event occurred. The overall rate of serious adverse effects was 0.0086%. In another study, Van Camp et al^19^ reported that the rate of SonoVue-related serious adverse effects was 0.014%. However, in a prospective study of 751 patients, 4.0% of the patients had side effects.^27^ Geleijnse et al^28^ reported that in their 352 consecutive cardiac SonoVue studies, 2.0% of the patients experienced mild or severe allergic reactions. We compared the intraprocedure adverse effects that occurred in patients who were not exposed to SonoVue with those patients who were exposed to SonoVue and showed that the HIFU-related adverse effects, such as leg pain, sacrum/buttock pain, groin pain, pain in the treated region, and discomfort on skin were more frequently observed in the patients who used SonoVue than those who did not use it. Leg pain, sacrum/buttock pain, and groin pain are correlated with the location of the lesions. However, we did not find a significant difference between the 2 groups in the locations of the lesions. These phenomena may be explained by that a small amount of SonoVue remained in the body which amplifyed the stimulation of surrounding nerves. We also observed that a few patients experienced increased blood pressure, nausea or vomiting, and bradycardia, but no significant difference was found between the 2 groups.

Recently, Papadopoulou et al^29^ performed a prospective study in 1010 children to evaluate the safety of SonoVue. Minor adverse events included dysuria, urinary retention, abdominal pain, anxiety, vomiting, perineal irritation, and urinary tract infection within 10 days after using SonoVue, and the rate of dysuria was 2.57%.^18^ In the present study, the rate of adverse effects was lower than the results from the study of Papadopoulou et al. We further compared the postprocedure adverse effects or complications between the patients who were exposed to SonoVue and those who were not. Except the higher rate of pain in the lower abdominal wall of the patients who were exposed to SonoVue during HIFU, we did not find any other significant difference between the 2 groups in HIFU-related adverse effects or complications (Table 5).

Our results demonstrated that SonoVue may play a role in the occurrence of acute renal function failure. In this study, 2 patients in the group who used SonoVue during HIFU had acute renal function failure. As no such case was reported when we did not use SonoVue to evaluate HIFU treatment efficacy over the last 10 years and no acute renal function failure occurred in the group who were not exposed to SonoVue in this study, this complication is likely related to SonoVue. We reviewed these 2 cases and found that one patient with a solitary fibroid was 49 years old. She had a history of hypertension for >10 years. During HIFU treatment, the patient complained of mild pain in the treated region and sacrum/buttock pain, but no other adverse effects occurred. On the first day after HIFU treatment, a significant rise in blood pressure (180/120 mm Hg) occurred, along with experiences of dizziness, headache, and vomiting. After administration of nitroglycerin, the blood pressure returned to the previous level. On the second day, the urine volume was just 450 ml for 24 hours and the patient appeared with dysphoria and disorientation. A blood test showed that the urea was at 16.50 mmol/L, whereas the creatinine was at 525.40 mmol/L. Continuous renal replacement therapy (CRRT) was given to the patient. Two weeks later, she recovered from this incidence and was discharged from the hospital when the urine volume was >1500 mL/24 h, and the renal function returned to normal.

Another patient was 36 years old and had a single symptomatic uterine fibroid. She reported a history of taking nonsteroidal anti-inflammatory drugs (NSAIDs) for headache for the past 6 years. During HIFU treatment, the patient only complained of mild pain in the treated region and a skin “hot” sensation. Before and immediately after HIFU, intravenous injection of 2.0 mL SonoVue was used to assess the treatment efficacy. On the fifth day after HIFU, the patient reported dysuria and the creatinine level reached 520 μmol/L. She was hospitalized and treated with CRRT. Two weeks later, she was discharged after urine volume and creatinine both returned to normal levels. We do not exactly know why acute renal function failure occurred in this study. As all the patients had the specific bowel preparation before HIFU treatment for 3 days, the fasting, bowel cleansing, and enema may have caused dehydration; a history of hypertension and a history of taking NSAIDs combined together may tend to cause acute renal function failure. Therefore, for those patients who have a history of hypertension or taking NSAIDs for years, cautious usage of SonoVue is recommended during HIFU treatment.

The present study is limited. Because the patients were retrospectively analyzed from 5 centers, the skill level variability may have affected the results. Although the baseline characteristics of the 2 groups of patients had no significant differences, other unexpected factors might have played a role in affecting the results. This study is also limited because we only analyzed the patients with uterine fibroids and/or adenomyosis; therefore, the sample size is relatively small. These factors may also have contributed to the results. Therefore, to define the safety of intraprocedure CEUS during HIFU, future prospective studies with large sample size are required.

In summary, the results of this study demonstrated that SonoVue may increase the incidence rates of some common HIFU-related adverse consequences during HIFU treatment for benign uterine diseases, but most of which were acceptable and self-limiting. Based on our results, SonoVue could be safely used to assess HIFU treatment efficacy during HIFU. However, after HIFU treatment, renal function should be monitored in patients with a history of hypertension or taking NSAIDs.
